# SVD-CNN: A Convolutional Neural Network Model with Orthogonal Constraints Based on SVD for Context-Aware Citation Recommendation

**DOI:** 10.1155/2020/5343214

**Published:** 2020-10-22

**Authors:** Shaoyu Tao, Chaoyuan Shen, Li Zhu, Tao Dai

**Affiliations:** School of Software Engineering, Xi'an Jiaotong University, Xi'an, Shanxi 710049, China

## Abstract

Context-aware citation recommendation aims to automatically predict suitable citations for a given citation context, which is essentially helpful for researchers when writing scientific papers. In existing neural network-based approaches, overcorrelation in the weight matrix influences semantic similarity, which is a difficult problem to solve. In this paper, we propose a novel context-aware citation recommendation approach that can essentially improve the orthogonality of the weight matrix and explore more accurate citation patterns. We quantitatively show that the various reference patterns in the paper have interactional features that can significantly affect link prediction. We conduct experiments on the CiteSeer datasets. The results show that our model is superior to baseline models in all metrics.

## 1. Introduction

Citation recommendation for researchers to quickly find the appropriate relevant literature is a rapidly developing research area [1]. Among this area, context-aware citation recommendation is a particular type for predicting citations for a citation context [2]. The citation context is usually a few sentences before and after the place holder, such as “”. The key problem for context-aware citation recommendation is how to measure the similarity between the citation context and a specific scientific paper.

**Figure 1:**
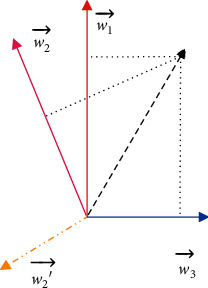
Distribution of the weight vector of the reference type in geometric space.

Similar to other NLP tasks (e.g., information retrieval (IR) and text mining), the simplest solution for context-aware citation recommendation calculates the relevant score between a citation context and candidate papers via Euclidean distance [3] and then selects the salient citations. However, simple text similarity is obviously too coarse to be a good measurement. In recent years, neural network models have been widely used to recommend documents due to their efficiency and effectiveness [4–7]. Neural network models can be regarded as better solutions than traditional machine learning methods for simplifying feature engineering tasks and having the ability to deal with large-scale data. However, the weight vectors in existing neural network-based models are usually strongly correlated. In fact, a critical assumption of using similarity measurements, such as Euclidean distance or cosine distance, is that the entries in the feature vectors should be possibly independent [8]. When the weight vectors are overcorrelated, some entries of the descriptor will dominate the measurement and cause poor ranking results. The above problems seriously affect the performance of citation recommendation because citing activity appears to have strong orthogonality. Assume there are three types of citations in a paper, including “field-reference” (red color), “method-reference” (purple color), and “math-reference” (blue color). “Field-reference” usually appears in the introduction and cites scientific articles that use the same techniques in other research fields. “Method-reference” usually appears in related work and cites scientific articles solving the same task. “Math-reference” usually appears in the main part of the paper describing the researcher's method in detail, and its citations will be more related to mathematical theorem. It is obvious that these three types of citations have strong orthogonality. In the neural network model, these three citation types are usually mapped into a matrix and can be seen as base vectors for inputs. As shown in Figure 1, vectors in the mapping matrix learned by traditional neural network models are not orthogonal. When a sample is mapped by $\overset{⟶}{w_{1}}$, $\overset{⟶}{w_{2}}$, and $\overset{⟶}{w_{3}}$, apparently $\overset{⟶}{w_{1}}$ and $\overset{⟶}{w_{3}}$ will dominate the output and consequently create low discriminative ability. A more satisfactory $\overset{⟶}{w_{2}^{\prime }}$ (yellow color) imposes orthogonality.

To address the aforementioned problems, we propose a neural network model with orthogonal regularization for context-aware citation recommendation. Our model uses CNN to extract the semantic features for citation context and candidate papers. We then add the orthogonal constraint based on SVD in our model to weaken the correlation of weight vectors in the FC layer, which can learn good interpretable features for citation context and papers. To the best of our knowledge, this is the first work that addresses the context-aware citation recommendation with the CNN and orthogonal constraint framework. Experimental results show that our model significantly outperforms other baseline methods.

## 2. Related Work

### 2.1. Citation Recommendation

A variety of citation recommendation approaches have been proposed in the literature, including text similarity-based [9, 10], topic model-based [11, 12], probabilistic model-based [13], translation model-based [7], and collaborative filtering-based [14]. Sun et al. [15] proposed a method for recommending appropriate papers for academic reviewers by using the similarity-based algorithm. Their method builds preference vectors for reviewers based on published history information and calculates the similarity between the preference vector and candidate document vector. The literature with high similarity is recommended to corresponding reviewers. Shaparenko and Joachims [16] considered the relevance of citation context and the paper content and applied a language model to the recommendation task. Strohman et al. [17] showed that using text similarity alone was not ideal for recommending citations, because scholars tend to construct new words to describe their own achievements, while two scholars who study the same topic may use different expressions for the same concept and method. To address this problem, Strohman et al. [17] regarded the document as a node in a directed graph to perform citation recommendations. They believe that the similarity measurement with reference information can reflect the reference situation of a node more authentically. Livne et al. [18] proposed a citation recommendation method by coupling the enriched citation context of the literature and adopted various techniques, including machine learning when making recommendations. Some works addressed the language gap between cited papers and citation contexts and attempted to use translation models or distributed semantic representations. Lu et al. [19] assumed that the languages used in the citation contexts and in the cited papers were different and used a translation model to solve this problem. He et al. [3] combined a language model, topic model, and feature model to find the appropriate citation context. Huang et al. [20] assumed that the appearance of cited papers was a particular language and represented the cited papers in unique IDs regarded as new “words.” The probability of citing a paper given a citation context is directly estimated by using a translation model. Tang et al. [21] proposed a joint embedding model to learn a low-dimensional embedding space for both contexts and citations.

In recent years, neural networks have shown better performance in many fields. Some researchers have attempted to recommend citations by using neural networks. Huang et al. [4] learned a distributed word representation for citation context and associated document embedding via a feedforward neural network and then estimated the probability of citing a paper by a given citation context. Tan et al. [5] proposed a neural network method based on LSTM to solve quote recommended tasks. They focused on the characteristics of quotes and trained neural networks to bridge the language gap. A neural network model learned the semantic representations of arbitrary length texts from a large corpus.

### 2.2. Orthogonal Constraint in Deep Learning

One of the greatest advantages of orthogonal matrices is that the norm of the matrix is changed when it is multiplied by a matrix. This property is useful in gradient backpropagation, especially to deal with gradient explosion and gradient dissipation problems. Orthogonal regularization is widely used in many fields. Brock et al. [22] used orthogonal regularization to improve the generalization performance of image generation editor tasks by using generative adversarial networks (GANs) [23]. They further expanded their work into BigGAN [24]. The results in their work showed that by applying orthogonal regularization, the generator allows fine-tuning the tradeoff between fidelity and diversity of samples by truncating hidden spaces, which can make the model achieve the best performance in the image synthesis of class conditions. Another advantage of orthogonal matrices is that they benefit from deep representation learning. If the weight vectors of the full connection layer in the convolutional neural network are highly correlated, the individuals in each full-join description will also be highly correlated, which will highly reduce retrieval performance. Sun et al. [25] proposed SVD-Net to show that guaranteeing the feature weight of the FC layer can increase the orthogonal constraint of the network and improve the accuracy. Zheng et al. [26] reported that regularization was an efficient method for improving the generalization ability of deep CNN because it makes it possible to train more complex models while maintaining lower overfitting. Zheng et al. [26] proposed a method for optimizing the feature boundary of a deep CNN through a two-stage training step to reduce the overfitting problem. However, the mixed features learned from CNN potentially reduce the robustness of network models for identification or classification. To address this problem, Wang et al. [27] decomposed deep face features into two orthogonal components to represent age-related and identity-related features to learn the age-invariant deep face features. In the above model, age-invariant deep features can be effectively obtained to improve AIFR performance. Chen et al. [28] proposed a group orthogonal convolutional neural network (GoCNN) model based on the idea of learning different groups of convolutional functions that are “orthogonal” to those in other groups, i.e., with no significant correlation among the produced features. Optimizing orthogonality among convolutional functions reduces the redundancy and increases the diversity within the architecture. Moreover, it can also obtain a single CNN model with sufficient inherent diversity, such that the model learns more diverse representations and has stronger generalization ability than vanilla CNNs.

## 3. Proposed Method

### 3.1. Problem Formulation

**Figure 2:**
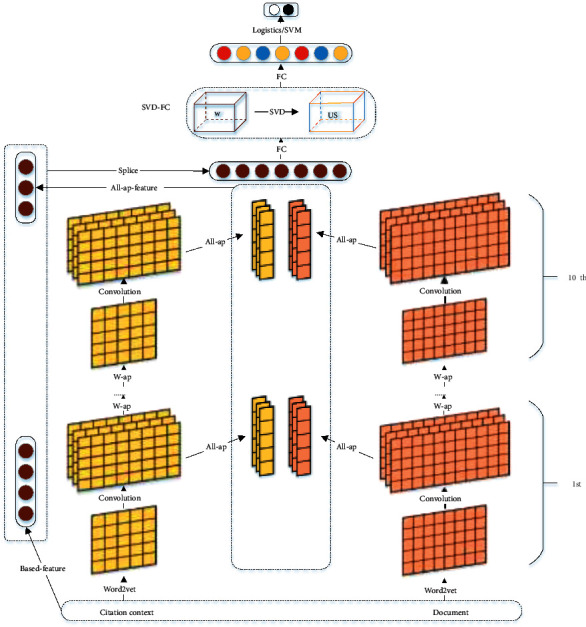
An overview of our model.

The context-aware citation recommendation is defined as the matching task between citation context and candidate papers. The main architecture of our model is shown in Figure 2. Our model is actually a convolutional neural network with two inputs and orthogonal constraints. Our model consists of the following main steps:

1. We adopt word2vec to obtain the raw input vectors and then use CNNs to extract multiple granularity semantic features
2. The multiple granularity semantic feature is then imposed orthogonally by an SVD-FC layer
3. We use fully connected layers to obtain the final vector representation. The logistic function or SVM is used to obtain the recommendation result

### 3.2. Network Structure

#### 3.2.1. Input Layer

Word2vec [29] is used to embed the input of our model. Each word is represented as a *d*_0_ dimensional precomputed vector, where *d*_0_=300. As a result, each sentence is represented as a feature matrix with dimension *d*_0_ × *s*. Through this layer, we can obtain the raw representation of citation context *c* and candidate document *d*.

We also calculate the weight of common words according to the inputs. Then, we can obtain the basic input features TF − IDF(*c*, *d*) for our model, which is the product of TF(*w*_*c*_, *d*) and IDF to reflect how important a word in citation context *c* is for a candidate document *d* in the corpus [30]. *w*_*c*_ is a word in citation context *c*. These two variables are calculated as follows:

(1) $$\begin{array}{c} \text{TF}\left(w_{c},d\right)=\frac{\text{count}\left(w_{c},d\right)}{\text{top}\left(w^{*},d\right)},\\ \text{IDF}=\log\frac{N}{\text{docs}\left(w_{c},D\right)}, \end{array}$$

where count(*w*_*c*_, *d*) is the number of words *w*_*c*_ that appear in document *d*. top(*w*^*∗*^, *d*) is the occurrence number of the word *w*^*∗*^ that appears most frequently in this candidate document *d*. docs(*w*_*c*_, *D*) is the number of documents containing the word *w*_*c*_ in all candidate citations *D*. *N* is the total number of candidate citations.

#### 3.2.2. Convolution Layer

**Figure 3:**
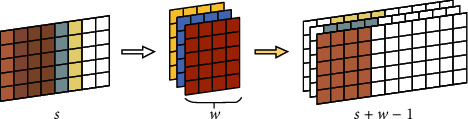
Convolution extraction generates phrases.

The inputs of the convolution layer are the feature matrix of citation context *c* and document *d*. The process of this layer is demonstrated in Figure 3. We first pad the two inputs to have the same length *s*=max(*c*, *d*) by zero vectors. For every input, let *v*_1_, *v*_2_,…, *v*_*s*_ be the words in a sentence. We define *g*_*i*_ ∈ *R*^*wd*_0_^, 0 < *i* < *s*+*w* − 1, as the concatenation of *v*_*i*−*w*_,…, *v*_*i*_. Then, this layer generates the feature *P*_*i*_ ∈ *R*^*d*_1_^ for the phrases *v*_*i*−*w*_,…, *v*_*i*_ as follows:

(2) $$\begin{array}{c} P_{i}=\tanh\left(W\cdot g_{i}+b\right), \end{array}$$

where *W* ∈ *R*^*d*_1_×*wd*_0_^ is a convolution kernel, and *b* ∈ *R*^*d*_1_^ is the bias.

#### 3.2.3. Average Pooling Layer

**Figure 4:**
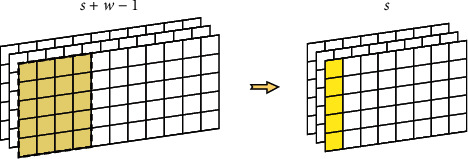
“*W*-ap” structure.

The pooling layer is usually used for feature compression. In our model, we choose average pooling. The reason is that whole sentences or paragraphs can express more meaningful semantics. As shown in Figure 4, we design two pooling layers. The first one is “*w*-ap,” which is the column average for the window of *w* continuous columns. After the convolution layer, an *s* column feature map is converted into a new *s*+*w* − 1 column feature map. By using “*w*-ap,” the new feature map is recovered into the *s* column. This architecture facilitates the extraction of more useful abstract features.

**Figure 5:**
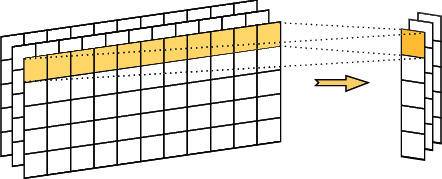
“All-ap” structure.

The second one is “all-ap,” which normalizes all columns. As shown in Figure 5, “all-ap” generates a representation vector for each feature map. The generated feature combines the information of the whole citation context or cited document.

**Figure 6:**
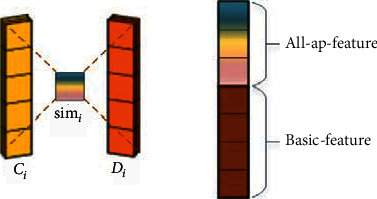
Generating the feature map.

Now, we can obtain the features of citation context and independent features of the cited document. The next step is to obtain the semantic relationships between the citation context and the candidate paper. We use cosine similarity to measure the semantic relations:

(3) $$\begin{array}{c} {\text{sim}}_{j}=\frac{\sum_{i=0}^{d_{j}}\left(C_{ji}\times D_{ji}\right)}{\sqrt{\sum_{i=0}^{d_{j}}{\left(C_{ji}\right)}^{2}\times \sum_{i=0}^{d_{j}}{\left(D_{ji}\right)}^{2}}},\;\left(j\in \left[1,10\right]\right), \end{array}$$

where *C*_*j*_ and *D*_*j*_ are the distributed representation of citation context and candidate document after the *j*‐th “all-ap” layer, respectively. A total of ten “all-ap” layers are carried out in our model. Therefore, *j* belongs to [1,10]. The benefit is that we can obtain the semantic relation between the citation context and the cited document with multiple granularities. As shown in Figure 6, the final output feature consists of all sim_*j*_ and basic features. Then, it is fed into the SVD-FC layer.

In most cases, we find that if we use all outputs of pool layers as the input of the SVD-FC layer, the performance will be improved. The reason is that features from different layers represent the different levels of semantics. Neglecting any layers will obviously cause information loss problems.

Next, we use the SVD-FC layer to learn the nonlinear combination features of citation relationships. This layer can force vectors in the feature map independent and orthogonal to each other. The added SVD-FC layer can also reduce the negative impact of excessive parameters.

#### 3.2.4. SVD-FC Layer

In this layer, we use SVD to factorize the weight matrix *W*(*W*=*USV*^*T*^) and replace it with *US*. Our experimental results show that replacing operations can reduce the negative impact on the sample space.

The Euclidean distance between samples can be used to measure whether their feature expression changes in a sample space. Denoting *e*_*m*_ and *e*_*n*_ as the feature maps of two different samples, we can obtain two different outputs of the full connection operation by using the weight matrix *W* or *US* as follows:

(4) $$\begin{array}{c} p=e\times W, \end{array}$$

(5) $$\begin{array}{c} q=e\times US. \end{array}$$

As seen in the above equations, *q* is orthogonalized output, while *p* is unorthogonalized. Then, we can obtain the following theorem.

**Theorem 1 .**

*p* and *q* in equations (4) and (5) will generate the same Euclidean distance for samples *e*_*m*_ and *e*_*n*_.

**Proof**

The Euclidean distance *L* between *p*_*m*_ and *p*_*n*_ is calculated as follows:

(6) $$\begin{array}{c} L={\left\|\overset{⟶}{p_{m}}-\overset{⟶}{p_{n}}\right\|}_{2}\\ =\sqrt{{\left(\overset{⟶}{e_{m}}-\overset{⟶}{e_{n}}\right)}^{T}WW^{T}\left(\overset{⟶}{e_{m}}-\overset{⟶}{e_{n}}\right)}\\ =\sqrt{{\left(\overset{⟶}{e_{m}}-\overset{⟶}{e_{n}}\right)}^{T}USVV^{T}S^{T}U^{T}\left(\overset{⟶}{e_{m}}-\overset{⟶}{e_{n}}\right).} \end{array}$$

Since *V* is an orthogonal matrix, equation (6) is equivalent to

(7) $$\begin{array}{c} L=\sqrt{{\left(\overset{⟶}{e_{m}}-\overset{⟶}{e_{n}}\right)}^{T}USS^{T}U^{T}\left(\overset{⟶}{e_{m}}-\overset{⟶}{e_{n}}\right)}\\ =\sqrt{{\left(\overset{⟶}{q_{m}}-\overset{⟶}{q_{n}}\right)}^{T}\left(\overset{⟶}{q_{m}}-\overset{⟶}{q_{n}}\right)}\\ ={\left\|\overset{⟶}{q_{m}}-\overset{⟶}{q_{n}}\right\|}_{2}. \end{array}$$

It can be seen that ${\left\|\overset{⟶}{p_{m}}-\overset{⟶}{p_{n}}\right\|}_{2}={\left\|\overset{⟶}{q_{m}}-\overset{⟶}{q_{n}}\right\|}_{2}$.

**Figure 7:**
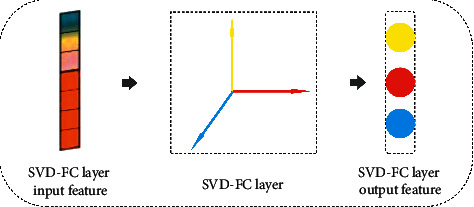
SVD-FC layer.

It should be noted that there are no negative impacts and no changes in discrimination ability for the entire sample space when replacing the weight. As shown in Figure 7, we use SVD of weight matrix *W* to map the feature map to an orthogonal linear space.

#### 3.2.5. Output Layer

The citation recommendation problem is regarded as a classification task in our model. In this layer, logistics and SVM can deal with binary classification tasks and predict the final citation relationship.

### 3.3. Training Details

#### 3.3.1. Embeddings

In our model, words are initialized by 300-dimensional word2vec embeddings and will not change during training. A single randomly initialized embedding is created for all unknown words by uniform sampling from[−0.01, 0.01]. We employ AdaGrad [31] and L2 regularization. We introduce adversarial training [32] for embeddings to make the model more robust. The process is achieved by replacing the word vector *v* after word2vec embeddings using word vector with disturbing *v*^*∗*^:

(8) $$\begin{array}{c} v^{*}=v\times r_{\text{adv}}, \end{array}$$

where *r*_adv_ is the worst case of perturbation on the word vector. Goodfellow et al. [33] approximated this value by linearizing the loss function $\log\;p\left(y|x,\hat{\theta }\right)$ around *x*, where $\hat{\theta }$ is a constant set to the current parameters of our model, and it only participates in the calculation process of *r*_adv_ without a backpropagation algorithm. With the linear approximation and *L*_2_ norm constraint, the adversarial perturbation is

(9) $$\begin{array}{c} r_{\text{adv}}=-\in \frac{g}{{\left\|g\right\|}_{2}},\;\text{where }g=\nabla_{x}\log\;p\left(y|x,\hat{\theta }\right). \end{array}$$

This perturbation can be easily computed by using backpropagation in neural networks.

#### 3.3.2. Layerwise Training

In our training steps, we define conv-pooling block *b*_*t*_(*t* ≥ 2), which consists of a convolution layer and a pooling layer. Our network model is then assembled by the initialization block *b*_1_ that initializes using word2vec and (*n* − 1) conv-pooling blocks.

First, we train the conv-pooling block *b*_2_ after *b*_1_ is trained. On this basis, the next conv-pooling block *b*_3_ is created by keeping the previous block fixed. We repeat this procedure until all (*n* − 1) conv-pooling blocks are trained.

Second, the following semiorthogonal training procedure is used to train the whole network.

Semiorthogonal training (SOT): it is crucial to train SVD-CNN, which consists of the following three steps:

 
*Step 1*. Decompose the weight matrix by SVD, i.e., *W*=*USV*^*T*^. *W* is the weight matrix of the linear layer. *U* is the left-unitary matrix. *S* is the singular value matrix. *V* is the right-unitary matrix. After that, we replace *W* with *US*. Next, we take all eigenvectors of *US*(*US*)^*T*^ as weight vectors.
 
*Step 2*. The backbone model is fine-tuned by fixing the SVD-FC layer.
 
*Step 3*. The model keeps fine-tuning with the unfixed SVD-FC layer.

Step 1 can generate orthogonal weights, but the performance of prediction cannot be guaranteed. The reason is that over orthogonality will excessively punish synonymous sentences, which is apparently inappropriate. Therefore, we introduce Steps 2 and 3 to solve the above problem.

The inputs of SVD-FC are defined as *YY*=(*y*_1_, *y*_2_,…,*y*_*m*_)^*T*^. The outputs are defined as *OO*=(*o*_1_, *o*_2_,…,*o*_*m*_)^*T*^. The weight matrix is defined as *W*=(*w*_1_, *w*_2_,…,*w*_*m*_)^*T*^. The expected outputs are defined as *A*=(*a*_1_, *a*_2_,…,*a*_*m*_)^*T*^. The error function is defined as

(10) $$\begin{array}{c} E=\frac{1}{2}\sum_{k=1}^{l}{\left(a_{k}-o_{k}\right)}^{2}, \end{array}$$

where *o*_*k*_=*f*(∑_*j*=0_^*m*^*w*_*kj*_*y*_*j*_),  *k*=1,2,…, *l*. Then, *E* with respect to *o*_*k*_ is derived, and the outcome is

(11) $$\begin{array}{c} \frac{\partial E}{\partial o_{k}}=-\left(a_{k}-o_{k}\right). \end{array}$$

We utilize the gradient descent strategy to find the gradient of the error with respect to weights. The iterative update of weights is as follows:

(12) $$\begin{array}{c} \Delta w_{kj}=-\eta \frac{\partial E}{\partial w_{kj}}. \end{array}$$

We define an error signal *δ*_*k*_^*o*^=∂*E*/∂ net_*k*_. equation (12) is equivalent to

(13) $$\begin{array}{c} \Delta w_{kj}=-\eta \frac{\partial E}{\partial \;{\text{net}}_{k}}\frac{\partial \;{\text{net}}_{k}}{\partial w_{kj}}\\ =-\eta \delta_{k}^{o}\frac{\partial \;{\text{net}}_{k}}{\partial w_{kj}}. \end{array}$$

According to equation (11), *δ*_*k*_^*o*^=∂*E*/∂ net_*k*_ is equivalent to

(14) $$\begin{array}{c} \delta_{k}^{o}=-\frac{\partial E}{\partial o_{k}}\frac{\partial o_{k}}{\partial \;{\text{net}}_{k}}=-\frac{\partial E}{\partial o_{k}}f^{\prime }\left({\text{net}}_{k}\right)\\ =\frac{\partial E}{\partial o_{k}}o_{k}^{\prime }=-\left(d_{k}-o_{k}\right)o_{k}^{\prime }. \end{array}$$

We use the sigmoid *f*(*x*)=1/(1+*e*^*x*^) as the nonlinear function, so equation (13) is equivalent to

(15) $$\begin{array}{c} \Delta w_{kj}=-\eta \delta_{k}^{o}y_{j}=\eta \left(d_{k}-o_{k}\right)o_{k}\left(1-o_{k}\right)y_{j}. \end{array}$$

In Step 1, the weight matrix *W* is decomposed by SVD and replaced with *US*. *U*=(*q*_1_, *q*_2_,…,*q*_*m*_)^*T*^, and *S*=diag(*λ*_1_, *λ*_2_,…, *λ*_*m*_). Since *d*_*k*_ − *o*_*k*_ is given, we define that Loss=*d*_*k*_ − *o*_*k*_. As a result, equation (15) is equivalent to

(16) $$\begin{array}{c} \Delta w_{kj}=\eta \text{ Loss}\cdot \left[o_{k}-\text{sigmoid}{\left(y_{j}\sum q_{i}\lambda_{i}+B\right)}^{2}\right]y_{j}. \end{array}$$

*q*_*i*_ · *q*_*j*_=0,  *i* ≠ *j* are in the left-unitary matrix *U*, so the model operation is not affected by the nonorthogonal eigenvectors *q*_*i*_. This is the reason for excessively punishing synonymous sentences in Step 1. However, orthogonality has a positive effect on Δ*w*_*kj*_ in Step 2.

The purpose of SVD is to maintain the orthogonality of each weight vector in geometric space. When weight vectors are conditioned by orthogonal regularization, the relevancy between weight vectors decreases. We use the following methods in Step 3 to measure relevance:

(17) $$\begin{array}{c} H=W^{T}W\\ =\left[\begin{array}{ccc} {\overset{⟶}{w_{1}}}^{T}\overset{⟶}{w_{1}} & \cdots & {\overset{⟶}{w_{1}}}^{T}\overset{⟶}{w_{k}}\\ \vdots & \ddots & \vdots \\ {\overset{⟶}{w_{k}}}^{T}\overset{⟶}{w_{1}} & \cdots & {\overset{⟶}{w_{k}}}^{T}\overset{⟶}{w_{k}} \end{array}\right]\\ =\left[\begin{array}{ccc} h_{11} & \cdots & h_{1k}\\ \vdots & \ddots & \vdots \\ h_{k1} & \cdots & h_{kk} \end{array}\right], \end{array}$$

where *W* is a weight matrix that contains *k* weight vectors: *w*_*i*_ (*i*=1,…, *k*).*h*_*ij*_ (*i*, *j*=1,…, *k*) is the dot product of *w*_*i*_ and *w*_*j*_. Let us define *S*(*W*) as the correlation measurement of all column vectors in *W*:

(18) $$\begin{array}{c} S\left(W\right)=\frac{\sum_{i=1}^{k}h_{ii}}{\sum_{i=1}^{k}\sum_{j=1}^{k}\left|h_{ij}\right|}. \end{array}$$

When *W* is an orthogonal matrix, the value of *S*(*W*) is 1. When *i* ≠ *j*, *S*(*W*) obtains the minimum value (1/*k*). Therefore, we can see that the value of *S*(*W*) falls into [(1/*k*), 1]. As a result, when *S*(*W*) is close to 1/*k* or 0, the weight matrix will have high relevance.

### 3.4. Complexity Analysis

Assume that the training sample size is |*C*|, the average number of words in each citation context is |*c*|, *C*_*l*_ is the number of kernels in the *l*-th layer, and *w*is the size of the sliding window. For one convolution layer, the training complexity is *O*(*C*_*l*−1_ · *C*_*l*_ · *w* · (*s* − *w*+1)). The training complexity of one *w*-ap layer is *O*(*C*_*l*_^2^ · *w* · *s*). The training complexity of one all-ap layer is *O*(*C*_*l*_^2^ · (*s* − *w*+1)), which was improved by C. F. Van Loan [12], computing the eigenvalue for SVD matrix decomposition with *K* size takes *O*(*K*) on the way of JACOBI. Assume that the size of the weight matrix in the SVD-FC layer is*K*, and the channel of the input matrix is *C*_in_. The computational cost for the SVD-FC layer is *O*(2*K*^2^ · *C*_in_+*K*).

## 4. Experiment

### 4.1. Dataset

We use the CiteSeer dataset [34] to evaluate the performance of our model. The dataset was published by Huang et al. [4]. In this dataset, citation relationships are extracted by a pair of citation contexts and the abstracts of cited papers. A citation context includes the sentence where the citation placeholder appears and the sentences before and after the citation placeholder. Within each paper in the corpus, the 50 words before and 50 words after each citation reference are treated as the corresponding citation context (a discussion on the number of words can be found in [7]). Before word embedding, we also remove stop words from the contexts. To preserve the time-sensitive past/present/future tenses of verbs and the singular/plural styles of named entities, no stemming is done, but all words are transferred to lower-case. The training set contains 3,989,547 pairs of reference contexts and citations, and the test set contains 1,021,685 citation relations.

Following common practice in information retrieval (IR), we employ the following four evaluation metrics to evaluate recommendation results: recall, mean reciprocal rank (MRR), mean average precision (MAP), and normalized discounted cumulative gain (nDCG).

### 4.2. Evaluation Metric

For each query in the test set, we use the original set of references as the ground truth *R*_*g*_. Assume that the set of recommended citations is *R*_*r*_, and the correct recommendations are *R*_*g*_∩*R*_*r*_. Recall is defined as

(19) $$\begin{array}{c} \text{recall}=\frac{\left|R_{g}\cap R_{r}\right|}{R_{g}}. \end{array}$$

In our experiments, the number of recommended citations ranges from 1 to 10. Recall evaluation does not reveal the order of recommended references. To address this problem, we select the following two additional metrics.

For a query *q*, let rank_*q*_ be the rank of the first correct recommendation within the list. MRR [35] is defined as

(20) $$\begin{array}{c} \text{MRR}=\frac{1}{\left|Q\right|}\sum_{q\in Q}\frac{1}{{\text{rank}}_{q}}, \end{array}$$

where *Q* is the testing set. MRR reveals the average ranking of the first correct recommendation.

For each citation placeholder, we search the papers that may be referenced at this citation placeholder. Each retrieval model returns a ranked list of papers. Since there may be one or more references for one citation context, we use mean average precision (MAP) as the evaluation metric:

(21) $$\begin{array}{c} \text{MAP}\left(d_{1},\ldots ,d_{N}\right)=\frac{\sum_{i}\left(R\left(d_{i}\right)/i\right)\sum_{j\leq i}R\left(d_{j}\right)}{\sum_{i}R\left(d_{i}\right)}, \end{array}$$

where *R*(*d*_*i*_) is a binary function indicating whether document *d*_*i*_ is relevant or not. For our problem, the papers cited at the citation placeholder are considered relevant documents.

We use normalized discounted cumulative gain (NDCG) to measure the ranked recommendation list. The NDCG value of a ranking list at position *i* is calculated as

(22) $$\begin{array}{c} \text{NDCG}\left(d_{1},\ldots ,d_{N}\right)=\sum_{i}\frac{2^{\text{rel }\left(d_{i}\right)}-1}{\ln^{i+1}}, \end{array}$$

where rel (*d*_*i*_) is the 4-scale relevance of document *d*_*i*_ in the ranked list. We use the average cocited probability [2] of 〈*d*_*i*_, *d*^*∗*^〉 to weigh the citation relevance score of *d*_*i*_ to *d*^*∗*^(an original citation of the query). We report the average NDCG score over all testing documents.

### 4.3. Baseline Comparison

We choose the following methods for comparison.

Cite-PLSA-LDA (CP-LDA) [36]: we use the original implementation provided by the author. The number of topics is set to 60.

- *Restricted Boltzmann Machine (RBM-CS)* [37]. We train two layers of RBM-CS according to the suggestion of the author. We set the hidden layer size to 600.
- *Word2vec Model (W2V)* [29]. We use the word2vec model to learn words and document representations. The cited document is treated as a “word” (a document uses a unique marker when it is cited by different papers). The dimensions of the word and document vectors are set to *n*=300.
- *Neural Probabilistic Model (NPM)* [4]. We follow the original implementation. The dimensions of the word and document representation vector are set to *n*=600. For negative sampling, we set the number of negative samples *k*=10, where *k* is the number of noise words in the citation context. For noise contrast estimation, we set the number of noise samples *k*=1000.
- *Neural Citation Network (NCN)* [7]. In NCN, the gradient clipping is 5, the dropout probability is 0.2, and the recurrent layers are 2. The region sizes for the encoder are set to 4, 4, and 5, and the region sizes for the author network are set to 1 and 2.

**Figure 8:**
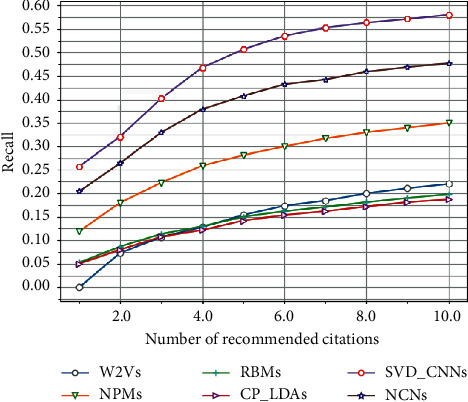
Comparison of recall with different methods on CiteSeer.

**Figure 9:**
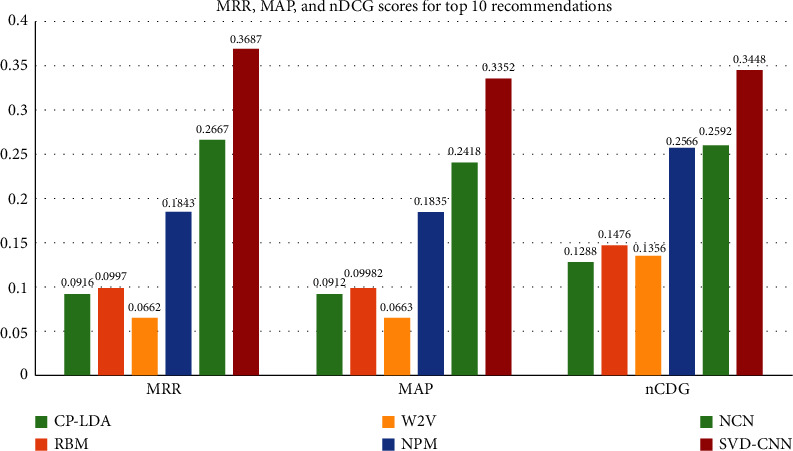
Comparison of MRR, MAP, and nDCG with different methods on CiteSeer.

Figures 8 and 9 show the performance of each method on the CiteSeer dataset. It is obvious that the SVD-FC model leads the performance in most cases. More detailed analyses are given as follows.

First, we perform a comparison among CP-LDA, RBM, W2V, and SVD-CNN. Our SVD-CNN completely and significantly exceeds other models in all metrics. The success of our model is ascribed to the content and correlation of our network. Due to the lack of citation context information, we find that W2V is obviously worse than other methods in terms of all metrics. CP-LDA works much better than W2V, which indicates that link information is very important for finding relevant papers. RBM-CS shows a clear performance gain over W2V because RBM-CS automatically discovers topical aspects of each paper based on citation context. However, the vector representations of citation context in RBM-CS are extracted by traditional word vector representations, which fully neglect semantic relations between the citation document and citation context and thus may be limited by vocabulary.

Second, we compare the performance among NPM, NCN, and SVD-CNN. It is not surprising that NPM and NCN achieve worse performance than SVD-CNN since their distributed representation of words and documents relies solely on deep learning without restraint. NPM recommends citations based on trained distributed representations. NCN further enhances the performance by considering author information and using a more sophisticated neural network architecture. However, the CNN in NCN does not have orthogonal constraints, which makes it difficult to capture different types of citing activities. In addition, NCN only utilizes the title of the cited paper for a decoder, which is apparently not sufficient for learning good embedding.

### 4.4. The Influence on the Link Prediction of Reference Pattern Interactional Features

According to the chapter positions of citation context in the article, we divide the training set into three parts: the introduction part contains 1,307,885 pairs of reference contexts and citations, the related word part contains 1,599,897 pairs of citations, and the main part contains 1,024,783 pairs. Furthermore, these datasets form three mixed datasets. In this part of the experiment, we use the CNN model without SVD as the baseline. These datasets are tested in a ratio of 3 : 1. In Tables 1 and 2, we show the results on the abovementioned datasets.

From the results, we obtain the following observations:

First, both CNN and SVD-CNN outperform unmixed datasets over mixed datasets across the different evaluation metrics, which shows that the diversity of reference patterns increases the difficulty of citation recommendation tasks.

Second, in Tables 1 and 2, we observe that our model is particularly good at resolving the difficulties in mixed datasets, which come from the diversity of reference patterns.

**Figure 10:**
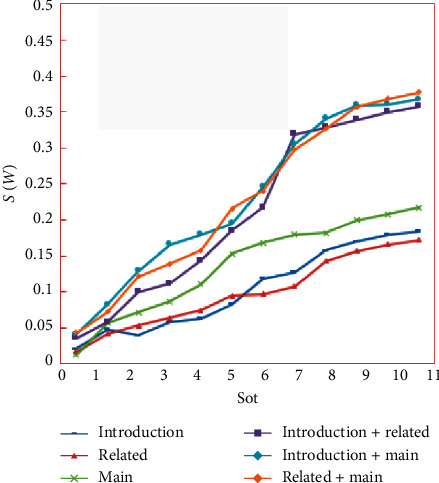
The change in *S*(*W*) during training on unmixed datasets and mixed datasets.

To better explore why mixed datasets are more complex than unmixed datasets, in Figure 10, we show the change in *S*(*W*) during the training process of SVD-CNN among various datasets.

As shown in Figure 10, the increase in *S*(*W*) on the mixed datasets indicates that SVD-CNN is good at decorrelation. We can also see in Tables 1 and 2 that the CNN model has pretty performance on unmixed datasets while achieving poor performance on mixed datasets. However, SVD-CNN achieves almost the same performance on the two types of datasets. This proves that the correlation from various reference patterns can significantly affect the link prediction.

The reason why the change in *S*(*W*) is not large on the unmixed datasets is that reference patterns of unmixed datasets have similar features, which belong to the same category. As a result, the orthogonality of the weight matrix is hard to improve on unmixed datasets. However, a citation recommendation algorithm has pretty performance on the unmixed datasets because there are low complexities.

Although mixed datasets are more complicated than unmixed datasets, SVD-CNN still performs well in mixed datasets. This indicates that SVD-CNN reduces the negative impact of the correlation of reference patterns, and our approach is more suitable for complex scenarios.

### 4.5. Comparison with Other Types of Decorrelation

In addition to SVD, there are still some other methods for decorrelating the feature matrix. However, these methods cannot maintain the discriminating ability of the CNN model. To illustrate this, we compare SVD with several varieties as follows:

1. Using the originally learned *W*
2. Replacing *W* with *US*
3. Replacing *W* with *U*
4. Replacing *W* with *UV*^*T*^
5. Replacing *W*with *Q* *D*, where *D* is the diagonal matrix extracted from the upper triangle matrix in *Q*-*R* decomposition
6. Replacing *W* with *W*^PCA^, where *W*^PCA^ is the diagonal matrix extracted from the weight matrix *W* after the processing of dimension reduction by PCA

After convergence of training, different orthogonal matrices are used to replace the weight matrix *W*. We define *T*-cost as the time cost of replacing the weight, which is equivalent to the proportion of the added time to the original time. As shown in Table 3, other types of decorrelation degrade the performance, in addition to *W*⟶*US* and *W*⟶*W*^PCA^. However, the time cost of *W*⟶*W*^PCA^ is more than that of *W*⟶*US*.

### 4.6. Ablation Study

In our method, there are two essential parameters, a term sot, which means the number of SOT iterations, and a biased parameter *d*_0_. In this section, we conduct an ablation study of these parameters.

**Figure 11:**
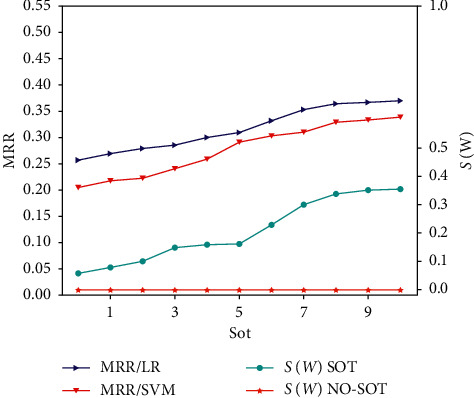
The performance impact of sot on CiteSeer.

We first evaluate the effectiveness of sot by empirically fixing *d*_0_=300. Since sot defines the loop time of orthogonal constraint training, it should be set as a nonnegative value.  Figure 11 illustrates the MRR with sot from 0 to 10 on the CiteSeer dataset. We can see that the performance improves as the value of sot increases. When sot=0, the model has no decorrelation and achieves the worst performance. In this situation, the weight matrix in the FC layer is highly correlated, and *S*(*W*) has the lowest value. The recommendation performance then increases while adding sot, which indicates that reducing the correlative degree of the weight matrix in the FC layer is critical for improving performance. When sot=10, our model achieves the best performance.

**Figure 12:**
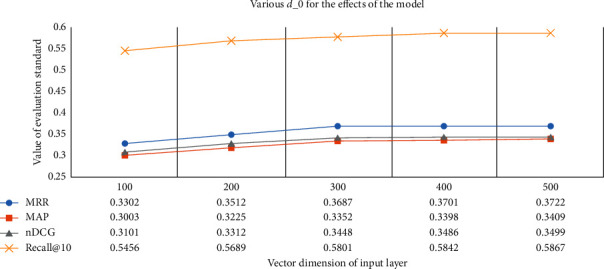
The performance impact of *d*_0_ on CiteSeer.

In our model, *d*_0_ is the dimension of citation context and cited document representations.  Figure 12 shows how the performance of SVD-CNN varies with *d*_0_ on the same sot. When *d*_0_ is small, the information content of the citation context is very small and produces worse performance. The recommendation performance increases to a maximum point until *d*_0_ reaches 300. It should be noted that although the larger *d*_0_ is better, the larger *d*_0_ will significantly increase the training time. Therefore, we choose *d*_0_=300.

## 5. Conclusion and Future Works

We propose a convolutional neural network model with orthogonal regularization to solve the context-aware citation recommendation task. In our model, orthogonal regularization is achieved by using SVD to factorize the weight of the FC layer, which can essentially make each vector in the feature map more independent. The orthogonal regularization also enhances the feature extraction ability of CNN. The experimental results show that SVD-CNN outperforms the other compared methods on CiteSeer. Our model only takes the abstract as the content of the cited paper. In the future, we will explore the performance of our model by using the full text of papers.

## Figures and Tables

**Table 1:** MRR metric on various datasets.

	Introduction	Related	Main	Introduction + related	Introduction + main	Related + main
CNN	0.3312	0.3294	0.3478	0.2773	0.2815	0.2978
SVD-CNN	**0.3995**	**0.4078**	**0.3989**	**0.3878**	**0.3889**	**0.3845**

**Table 2:** MAP metric on various datasets.

	Introduction	Related	Main	Introduction + related	Introduction + main	Related + main
CNN	0.3001	0.2909	0.3107	0.2572	0.2601	0.2637
SVD-CNN	**0.3701**	**0.3655**	**0.3693**	**0.3498**	**0.3511**	**0.3539**

**Table 3:** The comparison of related methods in Step 1.

	*W*	*W*⟶*US*	*W*⟶*U*	*W*⟶*UV*^*T*^	*W*⟶*Q* *D*	*W*⟶*W*^PCA^
Rank-1	63.6	63.6	61.7	61.7	61.6	63.6
mAP	39.0	39.0	37.1	37.1	37.3	39.0
*T*-cost	0%	36.27%	36.27%	36.27%	35.33%	57.65%

## Data Availability

Previously reported CiteSeer data were used to support this study and are available at [https://psu.app.box.com/v/refseer]. These prior datasets are cited at relevant places within the text as references [4].
